# Anti-band 3 and anti-spectrin antibodies are increased in *Plasmodium vivax* infection and are associated with anemia

**DOI:** 10.1038/s41598-018-27109-6

**Published:** 2018-06-08

**Authors:** Luiza Carvalho Mourão, Rodrigo de Paula Baptista, Zélia Barbosa de Almeida, Priscila Grynberg, Maíra Mazzoni Pucci, Thiago Castro-Gomes, Cor Jesus Fernandes Fontes, Sumit Rathore, Yagya D. Sharma, Rosiane A. da Silva-Pereira, Marcelo Porto Bemquerer, Érika Martins Braga

**Affiliations:** 1 Departamento de Parasitologia, Universidade Federal de Minas Gerais, Belo Horizonte, MG Brazil; 2 Center for Tropical and Emerging Global Diseases, University of Georgia, Athens, GA USA; 3 Embrapa Recursos Genéticos e Biotecnologia, Brasília, DF Brazil; 4 Centro de Pesquisas René Rachou, Fundação Oswaldo Cruz, Belo Horizonte, MG Brazil; 5 Faculdade de Ciências Médicas, Universidade Federal do Mato Grosso, Cuiabá, MT Brazil; 6 Department of Biotechnology, All India Institute of Medical Sciences, New Delhi, India

## Abstract

Clearance of non-infected red blood cells (nRBCs) is one of the main components of anemia associated with *Plasmodium vivax* malaria. Recently, we have shown that anemic patients with *P. vivax* infection had elevated levels of anti-RBCs antibodies, which could enhance *in vitro* phagocytosis of nRBCs and decrease their deformability. Using immunoproteomics, here we characterized erythrocytic antigens that are differentially recognized by autoantibodies from anemic and non-anemic patients with acute vivax malaria. Protein spots exclusively recognized by anemic *P. vivax*-infected patients were identified by mass spectrometry revealing band 3 and spectrin as the main targets. To confirm this finding, antibody responses against these specific proteins were assessed by ELISA. In addition, an inverse association between hemoglobin and anti-band 3 or anti-spectrin antibodies levels was found. Anemic patients had higher levels of IgG against both band 3 and spectrin than the non-anemic ones. To determine if these autoantibodies were elicited because of molecular mimicry, we used in silico analysis and identified *P. vivax* proteins that share homology with human RBC proteins such as spectrin, suggesting that infection drives autoimmune responses. These findings suggest that band 3 and spectrin are potential targets of autoantibodies that may be relevant for *P. vivax* malaria-associated anemia.

## Introduction

*Plasmodium vivax* accounts for a sizable portion of the global malaria burden and is being increasingly associated to fatal outcomes with anemia as one of the major complications^1,2^ particularly in young children^1–4^ and pregnant women^5,6^. Despite its enormous public health importance, the mechanisms behind vivax malaria-associated anemia are not well known.

Malaria-induced anemia is thought to arrise from hemolysis of infected RBCs, as well as from clearance of nRBCs^7,8^. Several reasons have been suggested to explain the removal of nRBC^8^, including impaired RBC production through dyserythropoiesis or bone marrow insufficiency^9^, exposition of erythrocytes to oxidative stress triggered by parasite rupture or host immune responses^10,11^, and mechanical *ex vivo* destruction of non-infected red blood cells (nRBCs), as it has been demonstrated using a splenic sinusoid model^12^, in addition to other mechanisms that may also be relevant to *P. vivax*-associated anemia. In *P. falciparum* infections, one of the causes underlying malarial anemia is the augmented removal of nRBCs possibly boosted by increased levels of self-antibodies against nRBCs proteins^13–15^. Evidences in this line are given by studies that have shown an inverse association between hemoglobin levels and anti-phosphatidylserine antibodies in humans with late post-anemia due to *P. falciparum* infection^14^. In addition, one may also take into account the expansion of T-bet+ B cells and the production of anti-erythrocyte antibodies in *ex vivo* cultures of naïve human peripheral blood mononuclear cells exposed to *P. falciparum*-infected erythrocyte^15^. On the other hand, the role of autoimmunoglobulins in *P. vivax* infection is an important field of research that has been mainly explored through clinical studies and case reports^16–18^. Our group recently demonstrated that *in vitro* erythrophagocytosis of nRBCs was mediated by anti-erythrocyte antibodies purified from anemic patients with vivax malaria, possibly through a decrease in cell deformability^19^. Thus, characterization of RBCs targets for autoimmunoglobulins elicited by *P. vivax* infection may be important for the understanding of vivax malaria-associated anemia as well as for autoimmune diseases due to its clinical and therapeutic potential. Herein we used two different strategies to understand the relationship between autoantibodies against nRBCs and *P. vivax*-associated anemia. First, we used an immunoproteomic approach to identify the erythrocytic antigens reactive to IgG from anemic *P. vivax*-infected patients. Then, we confirmed the reactivity of those antigens using ELISA and investigated the possible contribution of molecular mimicry to vivax malaria associated-anemia by searching for *P. vivax* proteins that share homology with human RBCs.

## Results

### Differential RBC protein recognition by IgGs from anemic and non-anemic *P. vivax*-infected patients with acute malaria

**Figure 1:**
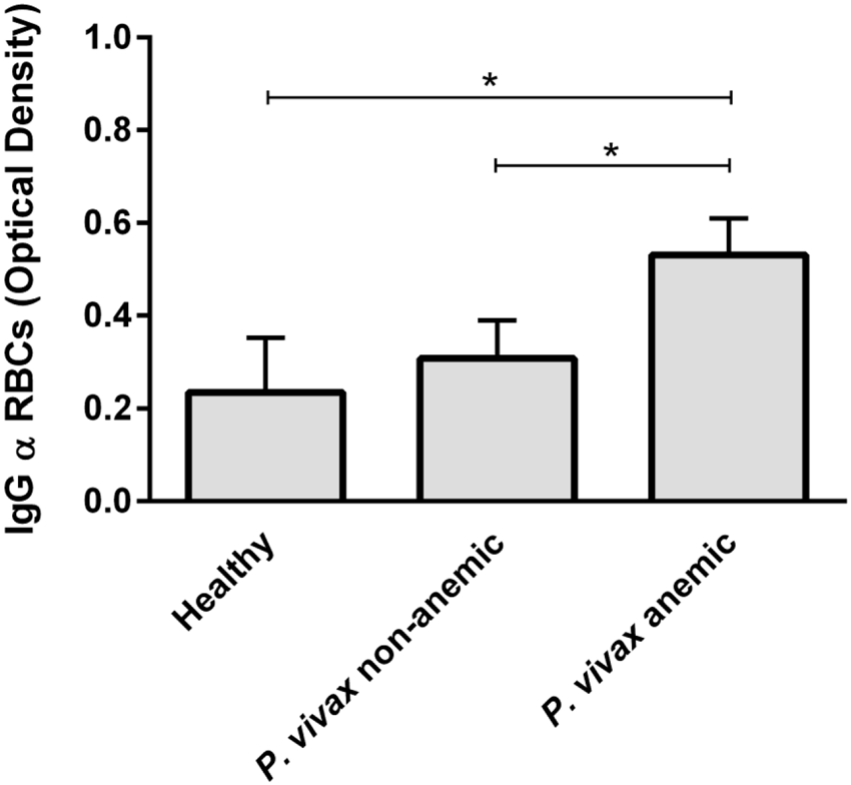
Levels of IgG against protein extracts of nRBCs in plasma from *P. vivax*-infected patients with or without anemia. IgG antibody responses were evaluated by ELISA using plasma from healthy individuals (n = 8) and anemic (n = 24) or non-anemic *P. vivax*-infected patients (n = 24). Results are shown as values of mean optical density and standard error of the mean. Differences between the groups were determined using Kruskal-Wallis test followed by Dunn post hoc test (p value <0.0001).

To determine whether IgG antibodies recognizing nRBC antigens are increased during acute *P. vivax* malaria, median levels of such immunoglobulins were assessed by ELISA in plasma from patients with patent *P. vivax* infection presenting (n = 24) or not (n = 24) anemia, as well as in plasma from healthy individuals never exposed to malaria (n = 8). Anemic patients infected with *P. vivax* had higher levels of IgG against erythrocyte proteins (median OD: 0.49; IQR [0.41–0.65]) than the infected non-anemic patients (median OD: 0.21; IQR [0.10–0.39]) or healthy controls (median OD: 0.21; IQR [0.07–0.37]) (Kruskal-Wallis followed Dunn’s post hoc test, p < 0.0001) (Fig. 1).

**Figure 2:**
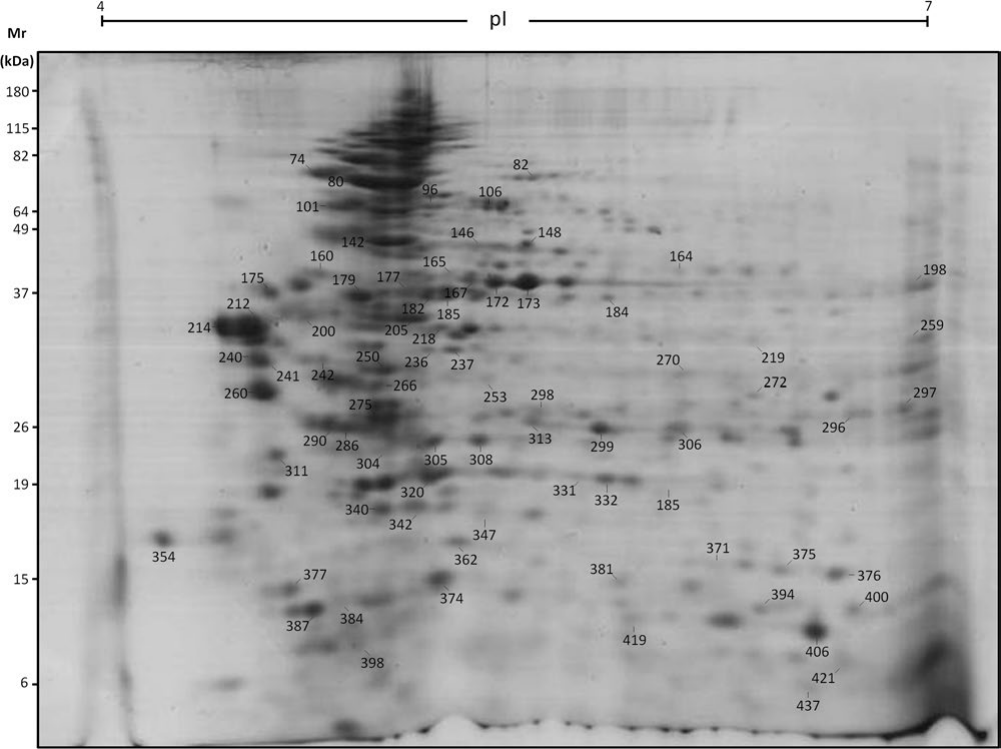
Representative 2D-SDS-PAGE map of RBC protein spots identified by mass spectrometry. 100 µg of nRBCs protein extract were focused on pH 4–7 IPG strips (7 cm) and then separated by SDS-PAGE 12%. Gel was stained with colloidal Coomassie Blue G-250. The molecular masses (kDa) of the protein standards are indicated on the left. All the protein spots that matched to their corresponding spot in the western blotting were excised from the gel and processed for MALDI-ToF/ToF mass spectrometry analysis. These protein spots are listed in the Supplementary Tables 1 and 2.

**Figure 3:**
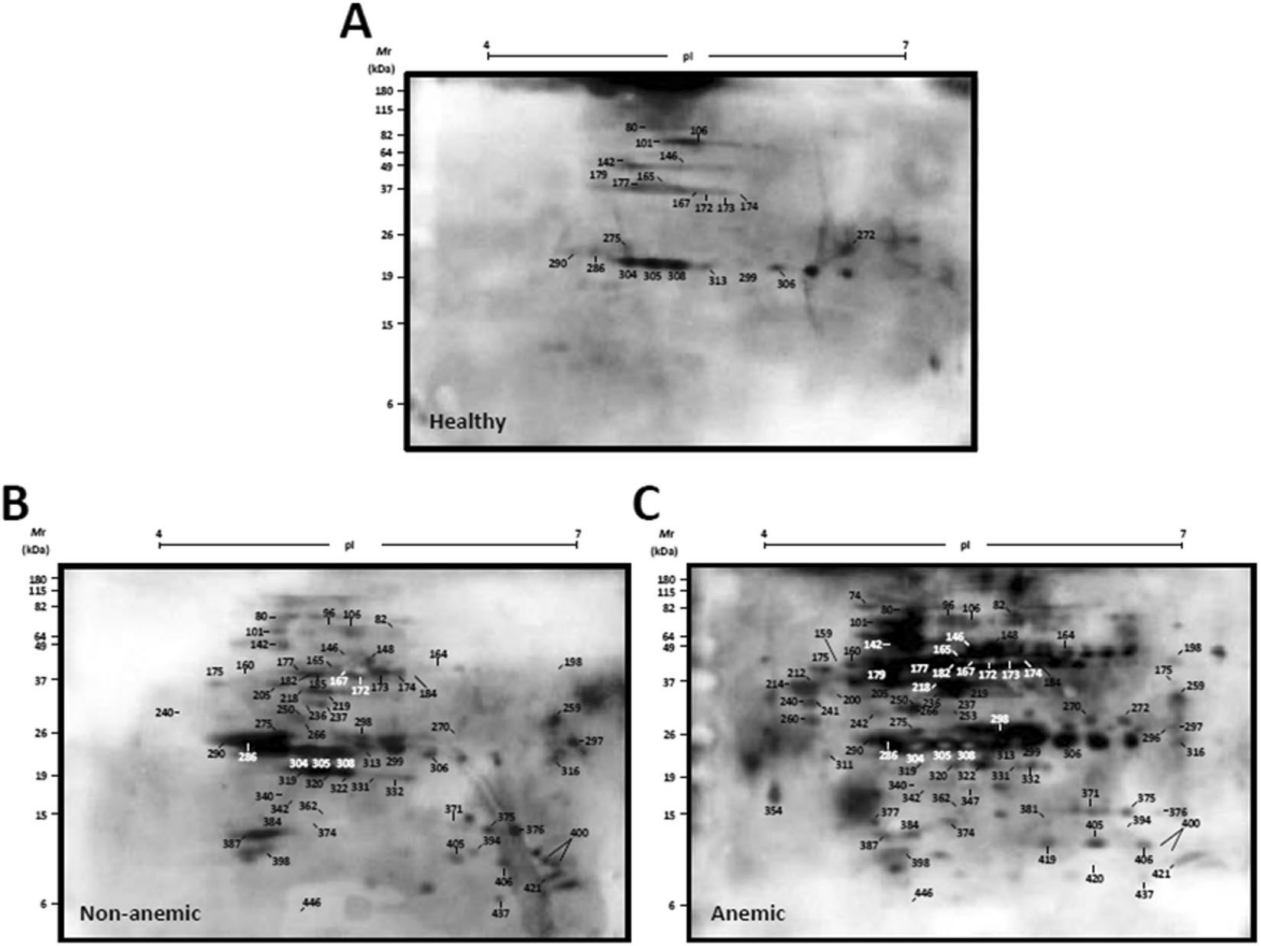
Comparative analysis of the IgG repertoire against nRBC proteins among subjects with distinct vivax malaria clinical features. The proteins resolved by 2D-SDS-PAGE were blotted onto PVDF membranes and probed with different plasma pools: (**A**) healthy, (**B**) patients with vivax malaria and no anemia or (**C**) *P. vivax*-infected patients with anemia. Bound antibodies were detected with HRP conjugated anti-human IgG (1:6000) using an ECL chemiluminescence-based kit. Images were analysed using ImageMaster 2D Platinum software (GE). A representative image of three independent experiments is shown. Western blots were cropped for easier visualization; uncropped images are available in the supplemental material. All the protein spots detected in the Western blotting that matched to their corresponding spot in 2D-SDS-PAGE gel were processed for MALDI-ToF/ToF mass spectrometry analysis and are listed in the supplementary Tables 1 and 2.

Comparative analysis of the immunoproteomes obtained with serum samples of the three experimental groups revealed a different profile of antigenic spots. Figure 2 shows a representative nRBC 2D-SDS-PAGE map. Sera from the infected groups recognized a greater number of spots than the sera from the healthy control group (Fig. 3). Some of the spots recognized exclusively by the serum antibodies of patients with vivax malaria corresponded to cytoplasmic proteins bound to the cell membrane such as ankyrin, dematin 7, and band 4.2 (Fig. 3, Supplementary Tables 1 and 2). Several spots corresponded to membrane-cytoskeleton associated proteins, spectrins and actin. Spots corresponding to the integral membrane protein band 3 were also identified (Fig. 3, Supplementary Tables 1 and 2). We also detected other proteins that were recognized by antibodies from *P. vivax*-infected patients, but which were not confirmed by peptide sequencing: EV15-like protein, kynurenine-oxoglutarate transaminase, long chain fatty acid transport protein 6, FAM45A protein, FAM180A, and TMLHE (Fig. 3, Supplementary Table 1). Spots recognized exclusively by sera from anemic malaria patients included band 3, spectrin (both alpha and beta chains), cytoplasmic actin 1, protein 4.1, protein band 4.2, ankyrin 1, and dematin (Fig. 3, Supplementary Table 1).

### Anti-band 3 and anti-spectrin antibodies correlate with anemia in patients with vivax malaria

Since band 3 and spectrin proteins are involved in RBC clearance, we selected them to validate the findings of the immunoproteomic approach. To detect anti-band 3 and anti-spectrin antibodies in plasma from patients with patent *P. vivax* infection, we used ELISA. IgG levels were expressed as reactivity index, which was obtained by dividing the mean OD value of each test sample by the cut-off value (threshold of positivity). The cut off value was calculated as the mean OD plus two standard deviations of eight healthy blood donors never exposed to malaria as it will be described in section 4.8.

**Figure 4:**
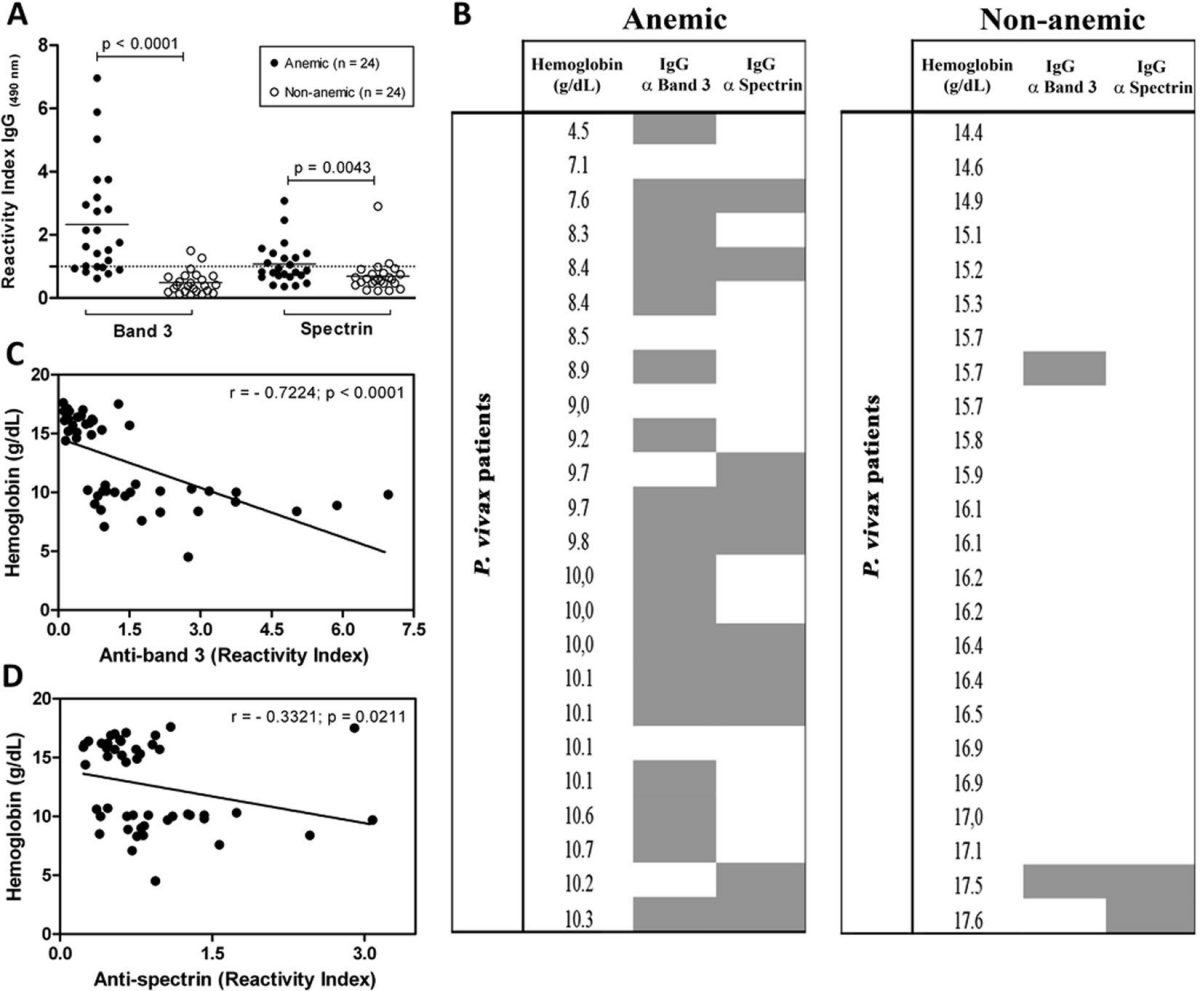
Anti-band 3 and anti-spectrin antibodies correlate with anemia in patients with acute *P. vivax* infection. Levels of anti-band 3 and anti-spectrin antibodies were detected by ELISA in plasma samples from patients with patent *P. vivax* infection presenting or not presenting anemia. Antibody levels were expressed as reactivity index (RI), which was calculated as the ratio between the mean OD generated by each test sample and the mean OD plus two standard deviations of samples from eight healthy blood donors never exposed to malaria. RIs equal or greater than 1.0 were scored as positive (**A**) Anti-band 3 and anti-spectrin antibodies levels were compared between anemic (n = 24) and non-anemic *P. vivax*-infected patients (n = 24) using Mann-Whitney test. Scatter plots show the means and standard deviations. The dotted lines on each graph represent the threshold above which samples were considered as positive. (**B**) Heat map of IgG antibody profile against band 3 and spectrin in plasma from anemic and non-anemic patients with vivax malaria. Grey colour indicates that antibody levels are at least greater than the mean plus two standard deviations of the healthy controls whereas white colour denotes the opposite result. Associations between hemoglobin levels and anti-band 3 or anti-spectrin antibodies (**C** and **D**, respectively) were analysed by Spearman correlation.

We observed that anemic patients had significantly higher levels of anti-band 3 antibodies (median: 1.69; ITR: [0.97–3.12]) in comparison to the non-anemic ones (median: 0.39; ITR: [0.21–0.69]) (Mann-Whitney U test p < 0.0001, Fig. 4A). A similar result was also observed for anti-spectrin antibodies (median of 0.85, ITR [0.68–1.38] for anemic *versus* median of 0.59, ITR [0.46–0.78] for the non-anemic; Mann-Whitney U test p = 0.0043).

To further understand each patient´s individual immunoreactivity profile against these two erythrocytic antigens, a heatmap was used (Fig. 4B). We observed that the spectra of antibody responses varied markedly among *P. vivax*-infected patients either presenting or not presenting anemia.

For evaluating whether there is a direct correlation between hemoglobin levels and anti-RBC antibodies, the levels of hemoglobin were correlated with the levels of anti-band 3 or anti-spectrin antibodies (Fig. 4C,D, respectively). The data clearly show that the levels of hemoglobin decreases with the increase in the level of anti-band 3 (Spearman correlation: r = −0.7224; p < 0.001). A similar association was also found to anti-spectrin antibodies (Spearman correlation: r = −0.3321; p = 0.0211).

### *In silico* identification of cross-reactive proteins between host RBCs and *P. vivax*

We hypothesized that the observed autoimmune response could be due to molecular mimicry. Therefore, we performed an *in silico* analysis comparing *P. vivax* entire proteome and human RBC proteome to identify putative molecular mimicry candidate proteins. The final BLAST results indicated that 23 *P. vivax* proteins were mimetic to human RBC proteins (Table 1). Nineteen of them were hypothetical proteins of the parasite and their mimetic human RBC proteins were associated with plasma membranes: ankyrin, actin, and spectrin (Table 1). The other four parasite proteins found to be mimetic to human RBC proteins (PVX_123515, PVX_099980, PVX_101610 and PVX_099980) were membrane surface-related proteins. Interestingly, most of the candidates were also membrane-associated RBC proteins such as the ones found in the immunoproteomic approach (Fig. 3, Supplementary Tables 1 and 2).

**Table 1:** *P. vivax* proteins mimetic to human proteins.

**Subject Human ID**	***P. vivax*** **query ID**	**Protein description (** ***P. vivax*** **)**	**Human protein**
sp|O60641|AP180_HUMAN	PVX_092010-AA:777	hypothetical protein, conserved	Clathrin coat assembly protein AP180
sp|O94856|NFASC_HUMAN	PVX_095335-AA:266	hypothetical protein, conserved	Neurofascin
sp|O94988|FA13A_HUMAN	PVX_123250-AA:290	hypothetical protein, conserved	Protein FAM13A
sp|P07205|PGK2_HUMAN	PVX_123515-AA:1081	MAC/Perforin domain containing protein	Phosphoglycerate kinase 2
sp|P35611|ADDA_HUMAN	PVX_099980-AA:800	major blood-stage surface antigen Pv200	Alpha-adducin
sp|P55209|NP1L1_HUMAN	PVX_091530-AA:524	hypothetical protein, conserved	Nucleosome assembly protein 1-like 1
sp|P78356|PI42B_HUMAN	PVX_099150-AA:363	hypothetical protein, conserved	Phosphatidylinositol 5-phosphate 4-kinase type-2 beta
sp|Q12873|CHD3_HUMAN	PVX_093655-AA:1234	hypothetical protein, conserved	Chromodomain-helicase-DNA-binding protein 3
sp|Q13151|ROA0_HUMAN	PVX_092895-AA:619	hypothetical protein, conserved	Heterogeneous nuclear ribonucleoprotein A0
sp|Q13637|RAB32_HUMAN	PVX_123100-AA:280	hypothetical protein, conserved	Ras-related protein Rab-32
sp|Q5S007|LRRK2_HUMAN	PVX_000660-AA:258	hypothetical protein	Leucine-rich repeat serine/threonine-protein kinase 2
sp|Q6PL18|ATAD2_HUMAN	PVX_101610-AA:137	RAD protein (Pv-fam-e)	ATPase family AAA domain-containing protein 2
sp|Q6WRI0|IGS10_HUMAN	PVX_085900-AA:1308	hypothetical protein, conserved	Immunoglobulin superfamily member 10
sp|Q6ZRI8|RHG36_HUMAN	PVX_114675-AA:140	hypothetical protein, conserved	Rho GTPase-activating protein 36
sp|Q9BQ39|DDX50_HUMAN	PVX_117850-AA:2285	hypothetical protein, conserved	ATP-dependent RNA helicase DDX50
sp|Q9BYB0|SHAN3_HUMAN	PVX_122920-AA:552	hypothetical protein, conserved	SH3 and multiple ankyrin repeat domains protein 3
sp|Q9BZF9|UACA_HUMAN	PVX_099150-AA:822	hypothetical protein, conserved	Uveal autoantigen with coiled-coil domains and ankyrin repeats
sp|Q9H254|SPTN4_HUMAN	PVX_003755-AA:415	hypothetical protein, conserved	Spectrin beta chain, brain 3
sp|Q9H4A3|WNK1_HUMAN	PVX_118355-AA:350	hypothetical protein	Serine/threonine-protein kinase WNK1
sp|Q9NQC3|RTN4_HUMAN	PVX_099980-AA:809	major blood-stage surface antigen Pv200	Reticulon-4
sp|Q9P0M6|H2AW_HUMAN	PVX_083455-AA:988	hypothetical protein, conserved	Core histone macro-H2A.2
sp|Q9UPE1|SRPK3_HUMAN	PVX_123910-AA:330	hypothetical protein, conserved	SRSF protein kinase 3
sp|Q9UPN3|MACF1_HUMAN	PVX_099150-AA:569	hypothetical protein, conserved	Microtubule-actin cross-linking factor 1, isoforms 1/2/3/5

### Comparison between the spectrin sequence of *P. vivax* and other *Plasmodium* species

**Figure 5:**
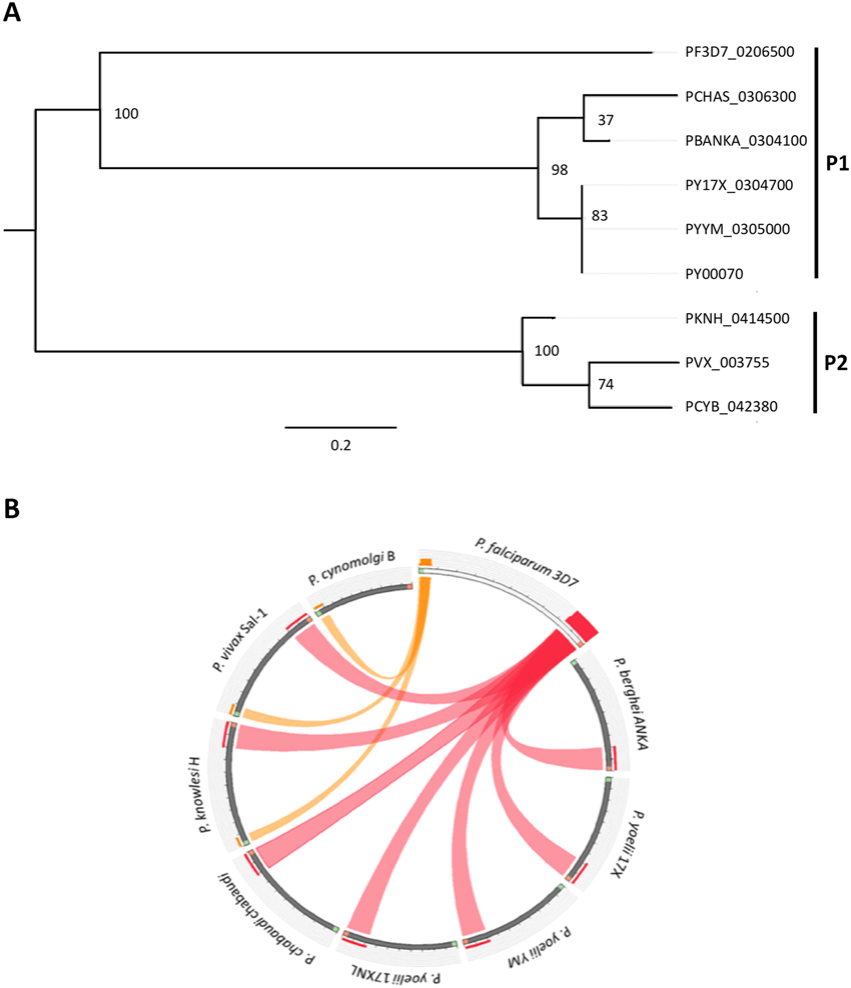
Comparison of the divergence of spectrin-like proteins between different *Plasmodium* species. (**A**) *Plasmodium* “spectrin-like” maximum likelihood phylogenetic tree. Two groups are detected: one group containing the *Plasmodium falciparum* protein (P1) and the other with the *P. vivax* protein (P2); P1 and P2 represents the two putative phylogenetic groups of spectrin-like sequences between the analyzed *Plasmodium* species. (**B**) *P. falciparum* protein Pf3D7_0206500 synteny alignment against all other *Plasmodium* species spectrin-like proteins. The figure shows that the C-terminus (red end) is highly conserved in all proteins and the N-terminus (green end) is only conserved in the group B sequences. Only alignments with more than 75% identity were represented in this figure. The conserved C-terminus region among all species is mimetic to the human pleckstrin-like homology domain (PH domain).

Since autoantibodies from *P. vivax*-infected patients recognized spectrin and this protein was identified in our mimicry *in silico* approach, we also verified if spectrin was conserved in other *Plasmodium* species. The sequence that seemed the most ideal for comparative analysis was the hypothetical *P. vivax* protein (PVX_003755), which was mimetic to the human spectrin β-chain. We aligned this *P. vivax* spectrin-like sequence with other *Plasmodium* species using blastp and verified that all local alignments corresponded to hypothetical proteins (Table 2). Our orthology analysis revealed no paralog sequences in the ortholog clusters. The phylogenetic analysis identified at least two evolutionary groups (P1 and P2) for this protein and showed that *P. falciparum* was the species whose spectrin sequence appeared to be the most divergent in comparison with the sequences from other *Plasmodium* species (Fig. 5A).

**Table 2:** *Plasmodium* spectrin-like ortholog sequences.

Gene	Organism	Product	Length
PBANKA_0304100	*Plasmodium berghei* ANKA	hypothetical protein, conserved	1030
PCHAS_0306300	*Plasmodium chabaudi chabaudi*	hypothetical protein, conserved	1027
PCYB_042380	*Plasmodium cynomolgi* strain B	hypothetical protein, conserved	840
PF3D7_0206500	*Plasmodium falciparum* 3D7	hypothetical protein, conserved	1436
PKNH_0414500	*Plasmodium knowlesi* strain H	hypothetical protein, conserved	1101
PY17X_0304700	*Plasmodium yoelii yoelii* 17X	hypothetical protein, conserved	983
PY00070	*Plasmodium yoelii yoelii* 17XNL	hypothetical protein	958
PYYM_0305000	*Plasmodium yoelii yoelii* YM	hypothetical protein, conserved	983
PVX_003755	*Plasmodium vivax* SaI-1	hypothetical protein, conserved	1085

We performed a sequence identity comparison of *P. falciparum* Pf3D7_0206500 against all other *Plasmodium* species spectrin-like proteins. One may observe two different patterns in our dataset: the C-terminus of all the proteins was highly conserved while the N-terminus region showed identity to *P. falciparum* only for proteins from the group P2 (Fig. 5B). When using all other *Plasmodium* sequences as queries we saw that they maintained the same identity pattern in the N-terminus except for *P. cynomolgi*, which seems to have a smaller size gene, caused by a mutation or by an annotation error.

## Discussion

Our data provide information about autoantibody responses to non-infected RBCs as well as its association with anemia during *P. vivax* infection. Using different approaches, we found that: (1) anemic patients infected with *P. vivax* have higher levels of IgG against erythrocyte proteins; (2) band 3 and spectrin proteins are the main molecular targets in nRBCs differentially recognized by IgGs from anemic patients with patent *P. vivax* infection; and (3) a *P. vivax* protein of unknown function is mimetic to human spectrin suggesting that molecular mimicry may contribute to autoantibody immune responses to nRBCs.

Many RBC protein spots were recognized by autoantibodies of *P. vivax*-infected patients rather than by autoantibodies from healthy sera. Previous results of our group revealed that non-infected RBC have reductions in their dynamic membrane fluctuations when incubated with autoantibodies from patients with *P. vivax* malaria^19^. The immunoproteomic data presented here show that antibodies from *P. vivax*-infected subjects recognize proteins such as band 3, spectrin and actin. Since the transmembrane band 3 exposes new antigens during *Plasmodium* infection^20,21^, it is a more probable target of autoantibodies than the cytoskeletal proteins. Shibuya *et al*. (2018) proposed a model for antibody assess to the cytoplasmic domain of band 3 in hereditary spherocytosis, which was attributed to the hydrolysis of spectrin and ankyrin. Their proposal is that conformational changes in the extracellular portion of band 3 protein observed in patients with hereditary spherocytosis would lead to the hydrolysis of spectrin and ankyrin in the intracellular milieu, which directly affects cytoskeleton dynamics, leading to RBC clearance. In this scenario, the binding of anti-band 3 autoantibodies to band 3 protein on the surface of RBC during *P. vivax* infection could induce similar intracellular changes, affecting cytoskeleton and leading to a higher clearance of erythrocytes.

Although the presence of autoantibodies against RBCs has been previously reported in vivax infection^16^, only recently we demonstrated that autoantibodies mediate erythrophagocytosis of nRBCs possibly through a decrease in cell deformability^19^.

IgG autoantibodies of anemic patients infected by *P. vivax* recognized a larger number of erythrocytic antigens when compared to non-anemic individuals. Some spots exclusively recognized by autoantibodies from anemic vivax patients were identified as band 3 protein, an anion transporter that mediates cell flexibility and shape maintenance^22^. In *Plasmodium* infections, band 3 acts as a host receptor for interaction with different merozoite proteins such as MSP-1^23^, MSP-9^24^, and tryptophan-rich antigens^25,26^. In endemic areas where *P. falciparum* is the prevalent species, band 3 antibodies seem to be protective because children with higher levels of these antibodies had lower parasitemia than nonimmune ones^27,28^. However, the role of autoantibodies against band 3 remains controversial and has never been studied in *P. vivax* infections. We speculate that such autoantibodies may contribute to anemia by interacting with new exposed epitopes followed by C3b deposition on nRBCs^21^, by promoting erythrocyte clearance^29^ and by changing cell membrane deformability^19^.

Band 3 has a cytoplasmic domain, which binds to different proteins involved in RBC structure and function, and a transmembrane domain, which catalyses the chloride and bicarbonate antiporter transport^22,30^. Also, while one-third of band 3 molecules binds to spectrin and becomes immobile, the other two-thirds freely diffuse into the lipidic bilayer^31^. It is known that band 3 pass through molecular conformation modifications during initial immune responses to malaria parasites, which leads to exposure of new epitopes^20,21^. Thus, since these modifications in band 3 are associated to hemichrome deposition and reactive oxygen species generation, they may persist after parasite clearance giving rise to changes in chloride/bicarbonate transporter activity^21^, thereby affecting RBC CO_2_ transport and contributing to anemia. Also, possibility of enzymatic hydrolysis of spectrin may be taken into account^32^.

Several mechanisms are involved in the production of self-reactive antibodies in malaria including polyclonal activation of B cells induced by parasite antigens^33^, dysregulation of B lymphocytes^33,34^ and stimulation of specific B lymphocytes by molecular mimicry^35^. To investigate whether molecular mimicry is underlying vivax malaria-associated anemia, we identified *P. vivax* proteins that share homology at the molecular level with proteins from human RBCs using bioinformatics tools. Homologous regions of primary structure were found between *P. vivax* and human spectrin, a result that may explain the increase in levels of anti-spectrin antibodies detected by ELISA in plasma of patients infected by *P. vivax*. In line with this idea is the study by Berti *et al*.^36^, who reported that a surge in anti-spectrin antibodies following the intraperitoneal injection of spectrin in rats affected the homeostasis of RBC by accelerating their elimination and stimulating erythropoiesis. It is possible that host spectrin and spectrin-homologous proteins derived from the parasite trigger the generation of autoantibodies that mediate the destruction of nRBCs. This anemic scenario leads to increased erythropoiesis, which, in turn, favours reticulocyte infection by *P. vivax*, thus supporting molecular mimicry as an advantageous host-parasite adaptation.

Although little is known about molecular mimicry in *P. vivax*, this mechanism has been proposed for other *Plasmodium* species^37^. The protein candidates include: *P. falciparum* merozoite surface protein 1 (PfMSP-1), which has human epidermal growth factor-like motifs^38^; the *P. falciparum* translationally controlled tumor protein (PfTCTP), which is homologous to the human histamine releasing factor^39^; and *P. falciparum* erythrocyte membrane protein 1 (PfEMP1), which shares motifs with human vitronectin^40^.

Phylogenetic analysis comparing different *Plasmodium* species showed two groups of spectrin-like proteins, with *P. falciparum* as an outlier. The C-terminus of spectrin-like *Plasmodium* orthologue proteins are highly conserved (Fig. 5). To find a possible biological relevance of spectrin, putative domains were predicted and, curiously, the C-terminus conserved region was predicted to be a pleckstrin homology-like domain (PH-like). Although such predictions do not define the protein function, it is known that increased expression of this domain family in humans is associated with the presence of anemia^41^. Moreover, it has been shown that pleckstrin-2, which contains two PH domains, plays a critical role in erythropoiesis^42^. Altogether, these data may explain how molecular mimicry of spectrin relates with the anemic status induced by *Plasmodium* infection.

Since spectrin is present on the intracellular side of the erythrocyte membrane, it remains unclear how anti-spectrin antibodies bind to a cytoplasmic protein. Although we do not have an explanation for such observation, this is not the first time that immunoglobulins directed towards an intracellular antigen have been detected. The reaction of anti-spectrin antibodies with RBCs has been previously described by studies investigating the clearance of aged erythrocytes^43,44^. We suggest that during *P. vivax* infection, the destruction of erythrocytes releases spectrin in the plasma, where it could bind to circulating anti-spectrin antibodies, thereby eliciting complement deposition onto these complexes. RBC engulfment by macrophages is affected by cell stiffness and molecular interactions in cell surface^45^. Therefore, deposition of those immunocomplexes on the surface of nRBCs through the complement receptor 1 (CR1) could decrease nRBCs deformability thus predisposing them to clearance by phagocytes and contributing to anemia in vivax malaria.

Immune response against cytoskeletal proteins may contribute to falciparum malaria^46,47^. Nevertheless, no studies are available for *P. vivax*-associated anemia, but since our immunoproteomic data suggest the presence of antibodies for band 3 and for associated cytoskeletal proteins, and also considering that antibodies for those proteins are involved in RBC clearance^29^, further investigation of contribution of these antibodies to the pathogenesis of vivax-associated anemia is necessary.

## Methods

### Ethics statement

This study was conducted according to the principles expressed in the Declaration of Helsinki and approved by the Ethics Committee of the National Information System on Research Ethics Involving Human Beings (SISNEP - CAAE01496013.8.0000.5149). All healthy donors and patients were anonymized, and they provided written informed consent for the collection of samples and subsequent analysis.

### Plasma samples

Blood samples were collected between February 2006 and January 2008 from adults with patent *P. vivax* infection who attended and were diagnosed at the Hospital Universitário Júlio Muller, in Cuiabá (Mato Grosso State, Brazil). *P. vivax* mono-infections were diagnosed by thick blood smear and further confirmed by nested PCR amplification of species-specific sequence of the 18s SSU rRNA gene of *Plasmodium*, as previously described^48^. The clinical history and demographic profile were obtained from all the subjects.

Individuals were assigned according to the results of complete blood count into two groups: (i) malaria patients without anemia (n = 24) and (ii) malaria patients with anemia (n = 24) (Table 3). Anemia was set as hemoglobin levels less than or equal to 11 g/dL and only patients with normocytic (mean corpuscular volume 80–96 fL) and normochromic (mean corpuscular hemoglobin concentration 32–36 g/dL) anemia were included. Patients presenting severe malnutrition or infections such as HIV or hepatitis were excluded from the study. As control, we included plasma from healthy volunteers who had never been exposed to malaria (n = 8).

**Table 3:** Baseline characteristics of the study population.

Characteristic	*P. vivax* anemic (n = 24)	*P. vivax* non-anemic (n = 24)	*p* value (Mann-Whitney test)
Age (years)	29.5 [27.7–41.2]	30.0 [24.0–42.0]	0.8954
Number of malaria previous episodes	2 [1–4]	3 [0–10]	0.8895
Parasitemia (parasites/µL)	7,275 [2,418–12,331]	2,375 [7,000–16,500]	0.0517
Hemoglobin (g/dL)	9.7 [8.4–10.1]	16.1 [15.4–16.8]	<0.0001
Hematocrit (%)	28.0 [25.0–29.4]	46.1 [45.4–48.08]	<0.0001
Platelets (cells/mm^3^)	101,000 [69,000–126,000]	160,500 [112,750–193,250]	0.0106
Leucocytes (cells/mm^3^)	4,900 [3,700–6,000]	5,390 [4,633–6,313]	0.2930

Values are shown as median and interquartile ranges.

For the immunoproteomics approach, we prepared a pool containing plasma from seven individuals for each studied group. These individuals were those who presented median levels of IgG against nRBCs detected by ELISA as it will be described below in section 3.4.

It is important to mention that blood samples from *P. vivax*-infected patients were collected on the onset of diagnosis, during the acute phase of infection and before starting treatment. The treatment consisted in chloroquine (150 mg/day, 3 days) associated with primaquine (15 mg/day, 7 days) at doses calculated per kilogram of weight, as proposed by the Brazilian Ministry of Health guidelines for *P. vivax* uncomplicated malaria therapy.

### RBC extract preparation

Erythrocyte membrane extract was prepared with RBCs obtained from a healthy O+ blood type donor after separation using Ficoll-Paque Plus (GE Healthcare, Pittsburgh, PA). Hemoglobin-free erythrocyte ghosts were obtained by hypotonic lysis as previously described^49^. Membrane proteins were solubilized in 8 M urea, 2 M thiourea, 4% w/v CHAPS, 0.0025% bromophenol blue, 65 mM DTT (Bio-Rad, Hercules, CA, USA), 1% v/v BioLyte 3–10 100X buffer (Bio-Rad) and precipitated using 2D Clean-Up Kit (GE Healthcare, Pittsburgh, PA). Protein concentration was estimated using Bradford assay.

### Detection of IgG against nRBCs proteins

Serum levels of IgG against nRBCs extracts were determined by ELISA. Briefly, each well of a 96-well flat-bottomed polystyrene microplate (Corning Incorporation, Corning, NY, USA) was coated with 0.1 ng of RBC protein extract in 0.1 M carbonate buffer, pH 9.6, and then incubated overnight at 4°C. After five washes with PBS containing 1% (w/v) bovine serum albumin (BSA), the plate was blocked with nonfat powdered milk for two hours at 37 °C. Next, plates were washed and incubated, in duplicate, with serum samples diluted 1:200 in PBS/BSA for 2 hours at 3 °C. Wells were rewashed and incubated with horseradish peroxidase conjugated polyclonal anti-human IgG diluted 1:8000 in PBS/BSA for 90 minutes at 37 °C. Finally, the plates were washed again, and the reaction was revealed using 0.5 mg/mL o-phenylenediamine dihydrochloride substrate in 0.05 M phosphate-citrate buffer, pH 5.0. The reaction was stopped with 3M H_2_SO_4_. Optical density (OD_492 nm_) was determined in a Spectra Max 250 microplate reader (Molecular Devices, San Jose, CA). The levels of specific IgG were expressed as OD_492nm_.

### Two-dimensional Western blotting and protein identification

RBC proteins (100 µg) were separated by 2D gel electrophoresis. RBC extract was loaded onto immobilized pH gradient (IPG) strips (7 cm, pH 4–7; Bio-Rad) and subjected to isoelectric focusing. Next, RBC proteins were reduced, alkylated and separated on 12% SDS-polyacrylamide (SDS-PAGE) gel^50^. To identify nRBCs antigens recognized by antibodies from the studied groups, two 2D-SDS-PAGE were performed in parallel to each group. The preparative 2D-SDS-PAGE gels were stained with Coomassie brilliant blue G-250 (BioRad, Hercules, CA, USA). The remaining 2D-SDS-PAGE gels were electroblotted onto PVDF membranes (BioRad) in transfer buffer (25 mM Tris-Base, 192 mM glycine, 20% methanol, pH 8.3). Each membrane was probed with each serum pool diluted 1:200 in TBS (20 mM Tris-HCl, 500 mM NaCl pH 7.5) containing 0.05% Tween-20 and 1% BSA. HRP-conjugated goat anti-human IgG (1:6000) and a chemiluminescence-based kit (ECL; GE Healthcare) was used to detect the immunoreactive spots. Three immunoblots were performed for each serum group. After image analysis (Image Master 2D Platinum 7.0, GE Healthcare), all immunoreactive spots that matched to their homologues in 2D-SDS-PAGE gels were excised, distained and digested with trypsin (Trypsin Gold, Mass Spectrometry Grade, Promega, Madison, WI, USA). The tryptic peptides obtained were extracted and had their volume decreased under reduced pressure. After a desalting step (C18-µZipTip, Merck-Millipore, Bilerica, USA), the peptides were dried, suspended in ultrapure water, spotted onto a MALDI target plate (BrukerDaltonics, Bilerica, MA, USA) and analysed using MALDI-ToF and MALDI-ToF/ToF (Autoflex Speed or Ultraflex III, BrukerDaltonics). MS and MS/MS spectra were acquired using Flex Control 2.0 (Bruker Daltonics). The external calibration MS mode was performed using Peptide Calibration Standard II (Bruker Daltonics) according to manufacturer instruction. Mass analysis ranged from m/z = 500 to m/z = 4000. The ToF operation was conducted in the reflected mode. All mass spectra were obtained in the positive ion reflector mode, using α-cyano-4-hydroxycinnamic acid as matrix. For the calibration of the MS/MS mode, parent ions from each of those peptides contained in the calibration mixture were selected and fragmented. The fragmentation was conducted using LIFT™ technology^51^. MS/MS spectra were also obtained in the positive mode. Theoretical values of [M + H]^+^ for each precursor ion were calculated by “Isotope-MS” Protein Prospector tool (http://prospector.ucsf.edu/prospector/mshome.htm). Mass spectra were edited and analysed using Flex Analysis 3.3 and Biotools 3.0 (Bruker Daltonics). The lists containing experimentally obtained m/z ratios were compared to theoretical values generated by *in silico* digestion of all human protein sequences deposited in SwissProt and NCBInr protein databases through Biotools v program. 3.0 (Bruker Daltonics) coupled to the Mascot v. 2.1 and 2.2. Protein identification was assigned by peptide mass fingerprint (PMF) and by MS/MS Ion Search using the search program Mascot. Searches were performed firstly against the SwissProt database and when it did not lead to identification, they were confronted against the NCBInr. To avoid random matches, identification was considered valid only for samples whose scores exceeded the identity or extensive homology threshold value that was calculated by MASCOT (p < 0.05). To confirm the identifications and to identify the proteins that did not present a significant score in those analyses, MS/MS spectra were interpreted by manual inspection taking into consideration the widely accepted mechanisms for the fragmentation of peptides as well as the mechanically favoured or disfavoured cleavage events^52,53^.

### Preparation of RBC antigens

#### Band 3 protein

To separate erythrocytes from leucocytes, freshly drawn blood collected in heparinized tube was layered on Histopaque and centrifuged at 1000 g for 20 min. Packed erythrocytes were washed three times with PBS. Hemoglobin-free erythrocyte membranes (ghosts) were prepared by osmotic lysis of the washed erythrocyte. The washed cells were hemolyzed in ten volumes of ice-cold 5 mM phosphate buffer (pH 8.0) containing 0.1 mM PMSF and incubated 30 min at 4 ºC. The ghosts were centrifuged at 20,000 g for 30 min. The supernatant was carefully aspirated without disturbing ghost pellet. This procedure was repeated until membranes were free of hemoglobin and the pellet become milky white. Then, the peripheral proteins were stripped from membrane by suspending it in ten volumes of ice-cold 2 mM EDTA (pH 12.0). Immediately after dilution, the membrane was pelleted by centrifugation at 48,000 g for 30 min at 4 ºC and washed three times with 5 mM phosphate buffer, (pH 8.0). The stripped membrane (15–20 mg protein/mL) was resuspended in 5 mM phosphate buffer, pH 8.0, and five volumes of 1% (v/v) C_12_E_8_ in 5mM phosphate buffer, pH 8.0 was added. This mixture was incubated at 4 ºC for 20 min and centrifuged at 48,000 g for 2 h at 4 ºC. Band 3 protein was purified by affinity chromatography on aminoethyl-agarose resin (ABT beads, Madrid, Spain). Amino ethyl-agarose was packed in column and equilibrated with 0.1% C_12_E_8_, 5 mM phosphate buffer (pH 8.0). The solubilized membrane protein was loaded onto the column and washed with one bed volume of 0.1% C_12_E_8_ 5 mM phosphate buffer (pH8.0). A linear gradient of 0–300 NaCl in 0.1% (v/v) C_12_E_8_ in 5 mM phosphate buffer, pH 8.0, of ten bed volume were applied to column. Band 3 was eluted at 100 mM NaCl and collected in fractions of 1 mL in sterile tubes. Concentration was determined by spectrophotometry and purity of the preparation was checked by 10% SDS-PAGE. Eluted protein was divided in fractions at 1 mg/mL in 5 mM phosphate buffer and lyophilized overnight.

#### Spectrin protein

Spectrin was extracted from RBCs as previously described with some modifications^54–56^. Briefly, blood was collected and centrifuged at 1000 g, for 20 minutes at 4 ºC in order to remove plasma and buffy coat. Packed RBCs were washed three times with 20 volumes of 5 mM sodium phosphate buffer (pH 8.0) containing 0.95% (w/v) NaCl and the cells were collected after each wash procedure by centrifugation at 4000 g for 10 minutes. The cells were then hemolyzed in 5 mM sodium phosphate, pH 8.0, and the membranes collected by centrifugation at 37000 g for 15 min. Membranes were washed in the same buffer until a pale yellow colour appears. The supernatant was removed, and the pellet was suspended in an equal volume of spectrin removal buffer (0.2 mM sodium phosphate, 0.1 mM EDTA, 0.2 mM dithiothreitol (DTT), 20 µg/mL phenylmethyl sulfonyl fluoride (PMSF), pH 8.0, 37 ºC for 20–60 minutes for the release of the dimeric spectrin. The tetrameric spectrin was purified with the same protocol but the incubation was performed at 4 ºC. Membranes were removed from the incubated sample by centrifugation at 27000 g for 15 minutes, and the supernatant was concentrated by dialysis against spectrin removal buffer. Then, vesicles were clarified by centrifugation (27000 X g, 15 minutes) and both dimeric and tetrameric spectrins were purified after concentration by 30% ammonium sulphate precipitation followed by isolation by gel filtration chromatography on a Superose 6 10/300 GL column equilibrated with 10 mM sodium phosphate (pH 7.5) containing 0.1 M NaCl, 5 mM EDTA, 1 mM DTT, and 0.3 mM sodium azide. Concentrations were determined spectrophotometrically, and purity was checked by 7.5% DS-PAGE under reducing condition. Before ELISA experiments, spectrin was extensively dialyzed against 5 mM phosphate to remove DTT and EDTA.

### Detection of anti-band 3 and anti-spectrin antibodies

For detection of anti-band 3 and anti-spectrin antibodies, ELISA was carried out as previously described with some modifications^57^. Briefly, plates were coated overnight at 4 ºC with 5 ng of band 3 or 50 ng of spectrin, blocked with 3% BSA, and incubated with sera samples diluted 1:100 in PBS containing 1% BSA (PBS.1% BSA) for 2 h at 37 ºC. Peroxidase conjugated anti-human IgG at a dilution of 1:1000 in PBS.1% BSA was added and the plates were incubated at 37 ºC for 90 min. Wells were developed with *O*-phenylenediamine substrate (benzene-1,2-diamine). Antibody values were expressed as reactivity index (RI), which was calculated as the ratio between the mean OD generated by each duplicate and the mean OD plus two standard deviations of samples from eight healthy blood donors never exposed to malaria. RIs equal or greater than 1.0 were scored as positive.

### Bioinformatics analyses

#### Mimicry candidate detection analysis

To identify molecular mimicry candidate proteins between the entire *P. vivax* proteome and human RBCs, we used a modified Perl version of a local pipeline created by Ludin *et al*.^40^. The term mimetic is refereed here as the display of any parasite protein whose primary structure resembles primary structures of the host and possibly confers a benefit to the parasite. Ludin´s pipeline works following several steps. First, a blastp search against free-living eukaryotic organisms unrelated to pathogenicity was performed to eliminate the generally conserved proteins^58^. Then, specific parasites sequences were masked for possible peptide signal sites using Phobius software^59^ and divided into 14 length size overlapping fragments, which were aligned against our free-living control proteomes to remove similar peptides. Finally, these 14-mers sequences parasite-specific were compared using blast alignment against the RBC human proteome. Only the high-scoring segment pairs (HSPs), e-value ≤ 10^−10^, from BLAST were selected as final mimicry candidates. Throughout all pipeline, redundancy control filters steps were used.

#### Comparison between *P. falciparum* and *P. vivax* sequences orthologous to spectrin

We searched for amino acid sequence differences that could help to identify the specific aspects that make *P. vivax*-associated anemia different from the one observed for *P. falciparum*. We aligned *P. vixax* mimetic protein sequences against *P. falciparum* sequences, using blastp algorithm. To evaluate the gene disposition over the genus *Plasmodium*, a search for orthologous sequences was done between all available *Plasmodium* species in PlasmoDB version 29^60^. The mimicry amino acid sequences were submitted to the OrthoMCL program^61^ using a total of nine isolates from different *Plasmodium* species: *P. falciparum* 3D7 (PF3D7_0206500). *P. berghei* ANKA (PBANKA_0304100), *P. yoelli yoelli* 17X (PY17X_0304700), *P. yoelli yoelli* YM (PMY_0305000), *P. yoelli yoelli* 17XNL (PY00070), *P. chabaudi chabaudi* (PCHAS_0306300), *P. knowlesi* strain H (PKNH_0414500), *P. vivax* Sal-1 (PVX_003755) and *P. cynomolgi* strain B (PCYB_042380), which were publicly available at this time. The recovered sequences were subjected to a maximum likelihood phylogenetic analysis using PhyML^62^ and visualized using Figtree program (http://tree.bio.ed.ac.uk/software/figtree/).

### Data Availability

All data generated or analysed during this study are included in this published article (and its Supplementary Information files).

## Electronic supplementary material

Original 2D-SDS-PAGE stained with colloidal Coomassie Blue G-250 of 100 µg of RBC protein extract using 7 cm, pH 4-7 IPG strip

RBCs immunoreactive proteins identified by peptide mass fingerprint (PMF)

RBCs immunoreactive proteins identified by MS/MS peptide fragmentation or by de novo sequencing
