# Supplementary material for: Anti-band 3 and anti-spectrin antibodies are increased in Plasmodium vivax infection and are associated with anemia
Source: Sci Rep. 2018 Jun 8;8:8762. doi: 10.1038/s41598-018-27109-6 (PMC5993813; doi:10.1038/s41598-018-27109-6)

**Anti-band3 and anti-spectrin antibodies are increased in *Plasmodium vivax* infection and are associated with anemia**

Luiza Carvalho Mourão^1^, Rodrigo de Paula Baptista^2^, Zélia Barbosa de Almeida^1^, Priscila Grynberg^3^, Maíra Mazzoni Pucci^4^, Thiago de Castro Gomes^1^, Cor Jesus Fernandes Fontes^5^, Sumit Rathore^6^, Yagya D Sharma^6^, Rosiane A. da Silva-Pereira^4^, Marcelo Porto Bemquerer^3^, Érika Martins Braga^1*^.

^1^Departamento de Parasitologia, Universidade Federal de Minas Gerais, Belo Horizonte, MG, Brazil.

^2^Center for Tropical and Emerging Global Diseases, University of Georgia, Athens, GA, USA.

^3^Embrapa Recursos Genéticos e Biotecnologia, Brasília, DF, Brazil.

^4^Centro de Pesquisas René Rachou, Fundação Oswaldo Cruz, Belo Horizonte, MG, Brazil.

^5^Faculdade de Ciências Médicas, Universidade Federal do Mato Grosso, Cuiabá, MT, Brazil.

^6^Department of Biotechnology, All India Institute of Medical Sciences,
New Delhi, India.

Corresponding author: Érika Martins Braga - Universidade Federal de Minas Gerais Av. Antônio Carlos 6627 Pampulha Belo Horizonte 31270-901 [embraga@icb.ufmg.br](mailto:embraga@icb.ufmg.br)

**Supplementary Information**

**Supplemental figure 1. Original 2D-SDS-PAGE stained with colloidal Coomassie Blue G-250 of 100 µg of RBC protein extract using 7 cm, pH 4-7 IPG strip.** The molecular masses (kDa) of the protein standard are indicated on the left.


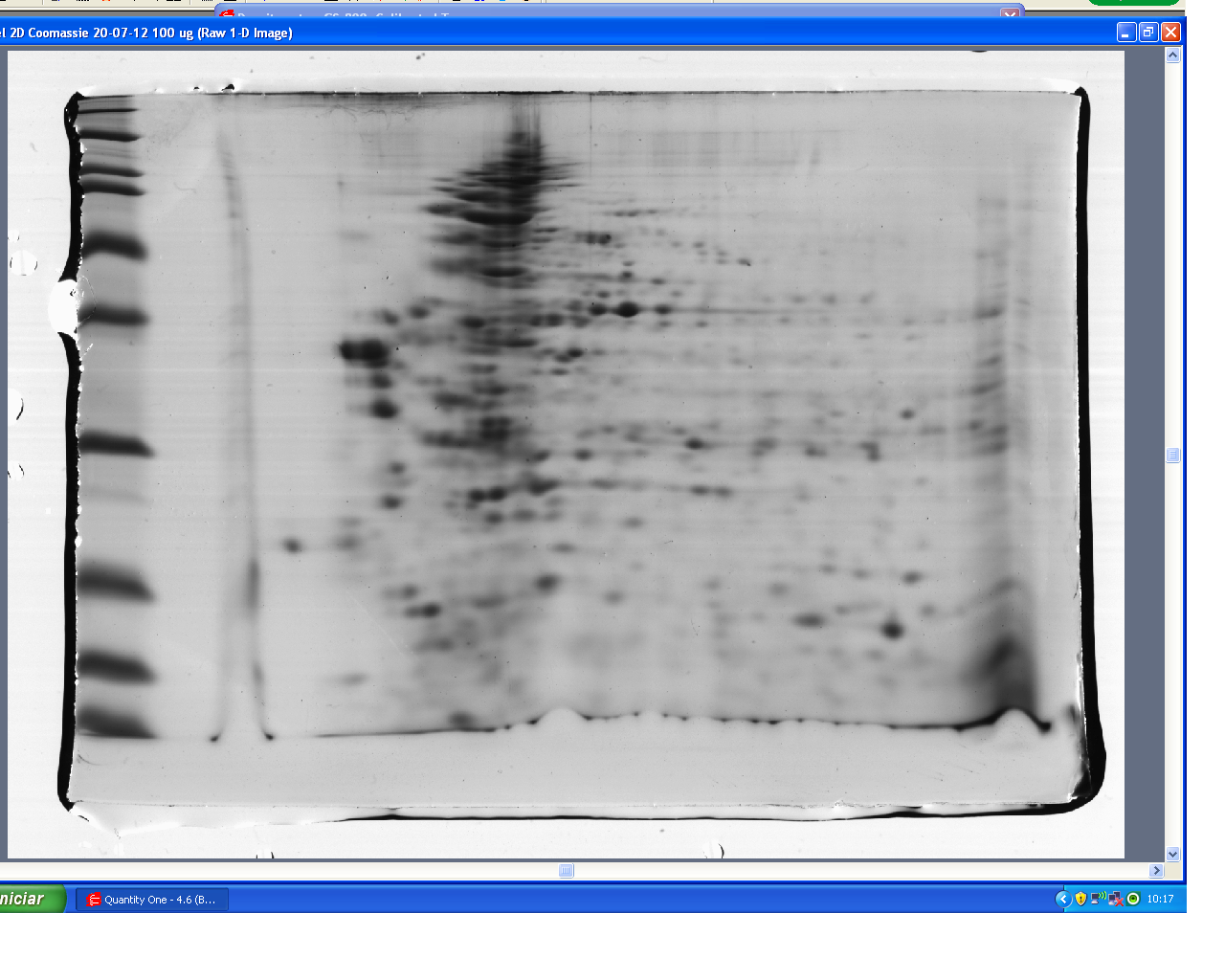


**Supplemental Figure 2: Western Blot for the 2D-SDS-PAGE gel probed with IgG purified from different plasma pools: (A) healthy, (B) patients with vivax malaria and no anemia, and (C) *P. vivax*-infected patients with anemia.** The molecular masses (kDa) of the protein standard are indicated on the left.

**A.**


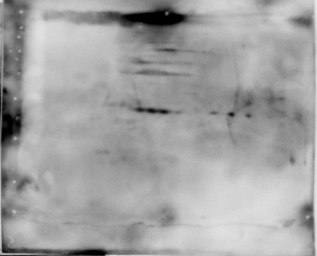


**B.**


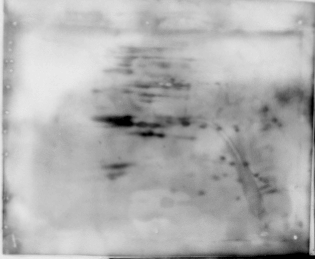


**C.**


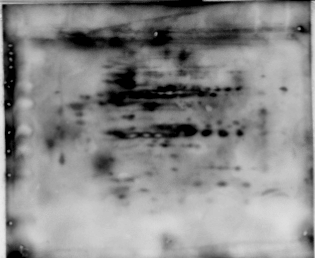

Supplement: Supplementary file 1 — Original 2D-SDS-PAGE stained with colloidal Coomassie Blue G-250 of 100 µg of RBC protein extract using 7 cm, pH 4-7 IPG strip [file 41598_2018_27109_MOESM1_ESM.doc]
